# David H. Hubel (1926–2013)

**DOI:** 10.1007/s00415-022-11153-4

**Published:** 2022-06-30

**Authors:** Stefano Sandrone

**Affiliations:** Department of Brain Sciences, Imperial College London, London, UK

**Fig. 1:**
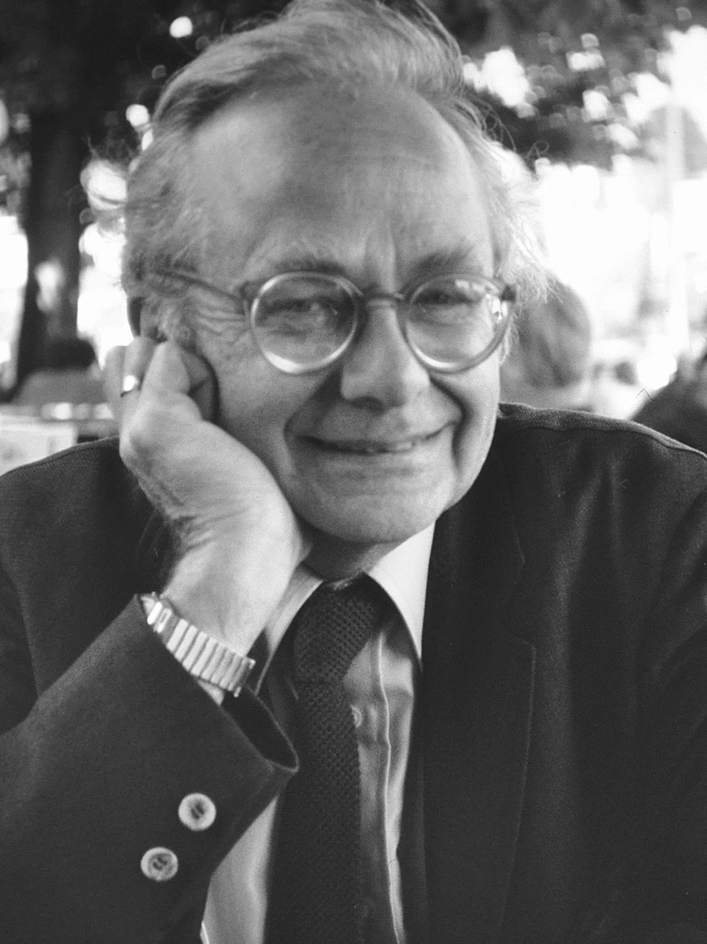
Portrait of David Hubel. Credits: Carl A. Hubel

David Hunter Hubel was one of the most outstanding neuroscientists of the twentieth century (Fig. 1). Born on the 27th of February 1926 in Windsor, Ontario, Canada, his parents were from Detroit, Michigan. They left the city to move to Canada (first Windsor, then Montreal) for his father’s job as a chemical engineer. From him, David inherited his interest in science, whereas his mother taught him to work hard to reach his goals [1]. Chemistry, electronics and music were his main hobbies [1–3]. Hubel attended the Strathcona Academy in Outremont and then McGill University, where he completed a bachelor’s degree in mathematics and physics in 1947 [4]. He was accepted at graduate work in physics and at the Medical School at McGill [5]: despite never taking a biology course [1], he opted for the latter.

After graduation in 1951, he did three years of hospital training plus a year of clinical neurophysiology at the Montreal General Hospital, under the mentorship of Herbert Jasper, for whom Hubel was reading electroencephalograms [5]. Both Herbert Jasper and Wilder Penfield prompted his fascination with the nervous system. Hubel married Ruth Izzard, a graduate of Donald Hebb’s psychology department [2], and was ready to start a new job as an assistant resident in neurology at Johns Hopkins. He was about to meet leading neurophysiologists Stephen Kuffler and Vernon Mountcastle, whose influence on his career would have been decisive [2].

After a year, he was drafted by the army and assigned to the Walter Reed Army Institute of Research, Neuropsychiatry Division. Hubel had no experience in electrophysiology or animal research [6] but could count on the neurophysiologist Michelangelo ‘Mike’ Fuortes as a mentor [6]. By displaying mechanical inventiveness and perseverance [7], Hubel developed a microdrive and a ‘varnish-insulated tungsten microelectrode’ [2, 8], which allowed him to record from single neurons in the visual cortex while marking the position of its tip by a microlesion [2]—instead of using glass pipettes and recording from axons, as many were doing [7].

It was in 1958 that Hubel started his collaboration with Torsten Wiesel, who had experience with retinal cell recording, having worked with Ken Brown [2]. Hubel and Wiesel were working within the lab of Stephen Kuffler, from whom they borrowed the experimental approach of finding simple ways to ‘isolate, insulate, amplify, visualize, record, or stimulate some part of the nervous system’ [7]. After hours of recording, long night experiments, and eleven internal drafts, epitomizing the couple’s perfectionism, they published their first paper, now widely considered a classic [3], in *Journal of Physiology* [9] in 1959, the same year that saw the entire laboratory moving to Harvard University. That paper was the beginning of one of the most successful collaborations in the history of neuroscience.

By performing experiments on cats (and then monkeys) with the microelectrode mentioned above, an advancer and an ophthalmoscope, Hubel and Wiesel explored a region that hardly anyone else was focusing on [7]. They were doing so with the spirit that moved Columbus to cross the Atlantic to see what could be found [3], as Hubel highlighted. The initial plan was to map the receptive fields of cells in the primary visual cortex of an anesthetized cat by projecting ‘images of light or dark spots directly onto the retina’ [2]. But while inserting the slide into the instrument to project the spot, they noted that neurons from the visual cortex produced a burst of activity, because of the line produced by the slide edge [5]. Upon changing the line’s orientation, the neuronal electrical activity changed too: different neurons had a preferred orientation, and all the orientations were represented in a neuronal sample [5]. Then, they differentiated between classes of visual neurons [6], discovered that neurons with similar functions were arranged together in columns [3], as shown by Vernon Mountcastle for the somatosensory cortex, and that, within a column, neurons preferred similar orientations [5]. They also revealed that columns of visual cortex neurons are dominated by one eye, that dominance is plastic and plasticity peaks during the ‘critical period’ [6] and championed the importance of curating strabismus in humans before the age of two [3].

Their collaboration, portrayed in the book *Brain and visual perception: the story of a 25-year collaboration* [10], culminated with the Nobel Prize in 1981 ‘for their discoveries concerning information processing in the visual system’; the other half was awarded to Roger Sperry for his studies on the functional specialization of the cerebral hemispheres [1]. Their findings laid the foundations for studies on higher brain functions [5], cortical plasticity and development [2].

Among the many other accolades, Hubel, who was the John Franklin Enders University Professor of Neurobiology, was elected to the National Academy of Sciences (1971), awarded the Louisa Gross Horwitz Prize from Columbia University (1978), was a founding member of the World Cultural Council (1981) and was elected as a Foreign Member of the Royal Society (1982). He won the Golden Plate Award from the American Academy of Achievement and the Ralph W. Gerard Prize in Neuroscience from the Society for Neuroscience (1993). He was also the President of the Society for Neuroscience between 1988 and 1989.

David Hubel was innovative, enthusiastic, and witty [5], yet worried by large laboratories headed by principal investigators too busy with administrative tasks and grant-writing to do their own experiments [4]. After becoming an emeritus professor, he ran a seminar on the fundamental of neurosciences and hands-on laboratory techniques for Harvard students [4] signed up by ten times as many students compared to the ones that could be accommodated [7].

On the 22nd of September 2013, seven months after his wife Ruth passed away, he died from kidney failure in Lincoln, Massachusetts, survived by three sons and four grandchildren. In addition to his breakthrough discoveries and beautifully crafted papers, David Hubel’s scientific legacy includes his passion for fundamental, discovery-based research [4], which he tirelessly championed with distinctive warm and humble manners [7].
